# Serum Carbohydrate Antigen 199 as a Biomarker for Evaluating Patients with Choledocholithiasis

**DOI:** 10.1155/2020/2739612

**Published:** 2020-03-25

**Authors:** Wang Gu, Zhong Tong

**Affiliations:** Anhui Medical University Third Affiliated Hospital, 390 Huaihe Road, Luyang District, Hefei, Anhui Province, China

## Abstract

**Aims:**

Choledocholithiasis is a common and yet potentially debilitating disease of the biliary tract. While certain patients with this disease remain largely asymptomatic or experience mild discomfort, in several cases, patient can suffer biliary inflammation and other serious symptoms. Previous studies have detected elevated serum levels of carbohydrate antigen 199 in patients with choledocholithiasis. We wanted to know whether serum CA199 level in patients with choledocholithiasis is related to the level of inflammation in patients.

**Methods:**

In the present study, we separated a cohort of 135 choledocholithiasis patients into two groups based on their white blood cell counts, which were either 3.5 − 9.5 × 10^9^/L or ≥9.5 × 10^9^/L. We also divided patients into two groups according to CPR < 10 mg/L and CRP ≥ 10 mg/L. At the same time, the correlation between CA199 and CRP level was analyzed.

**Results:**

We then used a Rank-sum test to compare serum carbohydrate 199 levels between these groups, revealing significantly higher levels of this antigen in patients with a white cell count ≥9.5 × 10^9^/L (*Z* = −3.584, *P* < 0.01). The two groups were grouped by CRP, and the CA199 level was compared. The difference between the two groups was statistically significant (*P* < 0.01). The correlation analysis between CA199 and CRP showed an obvious correlation (*r* = 0.574).

**Conclusion:**

This suggests that in patients with choledocholithiasis, higher circulating carbohydrate antigen 199 levels may correspond to a higher degree of inflammation.

## 1. Introduction

Choledocholithiasis is a common disease that has become increasingly frequent in recent years, with the most common treatments for this condition being either laparotomy or laparoscopic cholecystectomy and ERCP combined with endoscopic sphincterotomy [1–3]. If left untreated, the persistence of stones within the common bile duct can interfere with the normal excretion of bile, potentially leading to secondary acute cholangitis. While diagnostic strategies for detecting choledocholithiasis have improved significantly in recent years, there has not been a corresponding reduction in morbidity and mortality as a result of acute cholangitis secondary to choledocholithiasis. This is thought to be at least in part the consequence of a lack of satisfactory objective indices suitable for diagnosing acute cholangitis at an early stage. Previous studies have reported that elevated serum levels of carbohydrate antigen 199 (CA199) are detectable in patients with choledocholithiasis, rising to over 1000 U/ml in those patients suffering from cholangitis [4, 5]. We therefore hypothesized that these CA199 levels may be linked with the levels of inflammation in patients with choledocholithiasis, such that more severe inflammation may be associated with increased levels of circulating CA199. Levels of circulating white blood cells (WBCs) can be easily and cost-effectively measured in patients and can serve as an easily interpretable marker of systemic inflammation [6]. Elevated WBC counts often correspond to increased systemic inflammation, making them a potentially valuable diagnostic tool in the context of other findings [7]. As such, in the present study, we sought to determine whether WBC levels were correlated with CA199 levels in the serum of patients with choledocholithiasis, thus supporting a link between inflammation and levels of this putative circulating biomarker.

## 2. Patients and Methods

### 2.1. Patients

A total of 135 patients with choledocholithiasis that had been admitted to our hospital between 2016 and 2018 were enrolled in this study. Choledocholithiasis in these patients was confirmed via B-mode ultrasound, CT, magnetic resonance cholangiopancreatography, or intraoperative examination. On the day of admission, we obtained information pertaining to patient age, gender, C-reactive protein count, WBC count, CA99 levels, and total bilirubin levels.

### 2.2. Methods

(1) Grouping: 135 patients with choledocholithiasis were divided into two groups according to the CRP count. One group had a CRP count <10 mg/L, and the other group had a CRP count of ≥10 mg/L. We then used appropriate statistical tests to compare the CA199 levels between the two groups. (2) Examination items: the choledocholithiasis was confirmed by B-ultrasound, computed tomography, or MR cholangiopancreatography. Acute cholangitis is diagnosed as choledocholithiasis if Charcot's triple sign is found in these patients. Total DBIL and tumor marker carbohydrate antigen 199 (CA199), WBC, and CRP were detected in each patient. These tests were completed within 12 hours of admission. (3) The diagnostic methods of cholangitis: the diagnosis standard of acute cholangitis still depends on clinical manifestations and auxiliary examination [8]. (A) The clinical manifestations include ① history of biliary tract; ② fever and/or chills; ③ jaundice; and ④ abdominal pain (right upper abdomen or upper abdomen). (B) Laboratory data include evidence of inflammation and liver dysfunction. (C) Imaging findings include dilatation of the bile duct or etiological evidence (stenosis, stone, or stent).

### 2.3. Statistics and the Software of Statistics

SPSS 21.0 was used for all data analyses.

## 3. Results

Table 1 compiles the results of comparisons in gender, age, and total bilirubin between patient groups. No significant difference in gender was detected between groups as assessed via chi-squared test (*P* = 0.528 > 0.05). Similarly, no difference in age was detected between groups as assessed via an independent samples *t* test (*P* = 0.085 > 0.05). Total bilirubin levels in patients in this study were not normally distributed and were thus expressed as median (1st quartile, 3rd quartile). There was a significant difference in total bilirubin levels between the two groups, as determined via a rank sum (*P* = 0.017 < 0.05).

**Figure 1:**
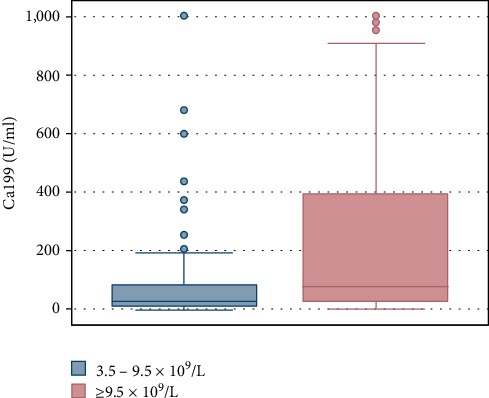


As CA199 levels were also nonnormally distributed, they too were expressed as median (1st quartile, 3rd quartile). Consistent with our hypothesis, there was a significant difference in these CA199 levels between groups as assessed via a rank sum test (*P* = 0.000 < 0.01). The results of these comparisons are shown in Table 2. The difference in CA199 levels between the two groups is also clearly visible in Figure 1. We further analyzed the correlation between WBC counts and CA199 levels, revealing a significant positive correlation between these two variables (*r* = 0.255; *P* < 0.01).

According to the results of c-reactive protein count grouping analysis, as shown in Table 3, the difference in CA199 level between the two groups was statistically significant (*P* < 0.01). The correlation between them was analyzed, and the results showed that there was an obvious correlation (*r* = 0.574, *P* < 0.01).

**Figure 2:**
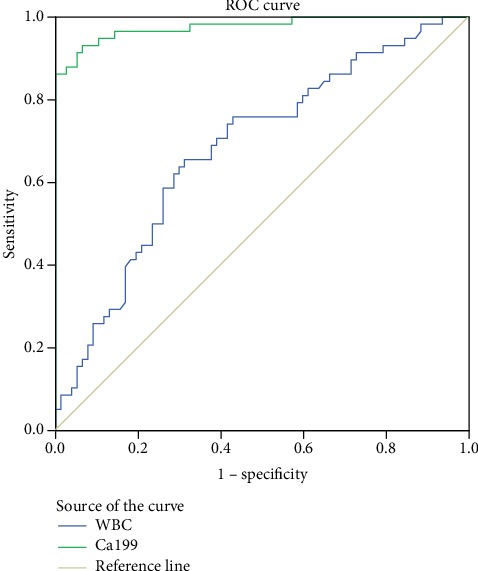


The area under the curve (AUC) of CA199 was 0.977 (95% CI: 0.953-1.000), and the 59.54 U/L threshold had the highest diagnostic accuracy with a sensitivity of 93.1% and specificity of 93.5%. The area under the curve (AUC) of WBC was 0.686 (95% CI: 0.594-0.775), and the 8.9 × 10^9^ threshold had the highest diagnostic accuracy with a sensitivity of 65.5% and specificity of 68.8% (Table 4 and Figure 2).

Factors with linear correlation in the correlation analysis were further introduced into the multiple linear regression equation, and the results showed that CA199 was related to WBC level, while age, gender, total bilirubin, and WBC had no linear relationship (all *P* values were >0.05) (Table 5). Factors with linear correlation in the correlation analysis were further introduced into the multiple linear regression equation, and the results showed that there was no linear relationship between CA199, age, gender, total bilirubin, and CRP (all *P* values were >0.05) (Table 6).

## 4. Discussion

CA199 is a carbohydrate antigen that is linked to the Lewis blood group antigen classification system. First discovered in human colorectal cancer cells, CA199 has since been found to be produced by a range of normal epithelial cell types, including those in the pancreas and the bile ducts [9]. In the current clinical practice, CA199 levels are commonly used as a biomarker of malignant tumors of the biliary tract and pancreas [10]. In most benign diseases, CA199 levels remain low, although levels in patients affected benign obstructive jaundice (including choledocholithiasis, cholangitis, and Mirizzi syndrome) are elevated [11, 12]. The mechanistic basis for such elevation is unclear. Proposed mechanisms include increased CA199 production in the bile duct as a consequence of increased biliary pressure leading to increased CA199 production either directly or indirectly as a consequence of inflammation. These changes ultimately increase CA199 production and/or enhance its release into circulation, allowing for it to be detected clinically. In addition, cholangitis is associated with inflammatory cytokine production [13].

Cholangitis is a form of biliary inflammation and infection that is secondary to obstruction, with choledocholithiasis being the most common cause of such obstructions. Infections in this context are most frequently caused by Escherichia coli, Klebsiella, Enterobacter, and Enterococcus. If untreated, cholangitis can rapidly progress to sepsis and endanger the life of affected individuals [14]. Symptoms of cholangitis include fever, jaundice, and right upper abdominal pain, which affect up to half of patients. In contrast, only about 5% of patients exhibit a combination of these symptoms together with psychiatric changes and hypotension indicative of severe sepsis [15]. The early identification and treatment of patients with choledocholithiasis are thus essential in order to prevent its progression to serious cholangitis. As the symptoms of this condition are nonspecific, cholangitis is typically based upon laboratory values and imaging studies, including elevated WBC and bilirubin levels [14].

It can be found from the ROC curve that CA119 and WBC have better sensitivity for the diagnosis of cholangitis, but the critical value of WBC is still in the normal physiological level, which may have limited significance for clinicians to make judgments, while CA199 is more sensitive, and the critical value is also outside the normal range, so it is more helpful for clinicians to make judgments. The correlation analysis results of customs clearance showed that CA119 had a certain correlation with WBC and CRP, but the correlation was not very strong. In addition, we conducted further linear multivariate analysis on WBC and CRP, and the results indicated that CA199 was an independent influencing factor for WBC, but had no significant correlation with CRP. The above conclusions can indicate that the level of CA199 represents the inflammatory level of patients to some extent, but the representativeness may not be so accurate, so comprehensive clinical analysis is needed, and CA119 can only be used as a part of the reference. Our results strongly suggest that CA199 levels can be used to gauge the risk of cholangitis secondary to choledocholithiasis and represent an ideal biomarker for identifying cholangitis in its early stages. Abnormally elevated CA199 levels may offer predictive value as a means of detecting acute cholangitis after choledocholithiasis, with CA199 serving as an inflammatory marker in the pathogenesis of this disease. Elevated Ca199 levels may thus suggest that doctors should treat this condition in order to prevent acute cholangitis onset, to alleviate patient pain, and to improve patient prognosis.

In conclusion, our results show that abnormally elevated levels of CA199 in the serum of patients with choledocholithiasis may be predictive of the risk of secondary acute cholangitis in these patients. Elevated CA199 levels should therefore alert clinicians to the possibility of patients developing acute cholangitis, allowing them to undertake appropriate interventions to prevent disease progression.

## Figures and Tables

**Table 1:** Contrast table of gender, age, and total bilirubin value in two groups.

WBC	*n*	Gender (male)	Age	TBIL
3.5~9.5 × 10^9^/L	74	36(48.65)	68.64 ± 14.56	27.75 (19.13,70.60)
≥9.5 × 10^9^/L	61	33(54.10)	72.69 ± 12.15	55.30 (26.85,110.80)
c^2^/t/z		0.397	-1.733	-2.390
*P*		0.528	0.085	0.017^∗^

^∗^ *P* < 0.05.

**Table 2:** Comparisons of Ca199 between the two groups.

WBC	*n*	Ca199	*z*	*P*
3.5~9.5 × 10^9^/L	74	28.71 (13.28,87.59)	-3.584	0.000^∗∗^
≥9.5 × 10^9^/L	61	78.89 (27.49,436.90)		

^∗∗^ *P* < 0.01.

**Table 3:** Statistic result of CRP.

CRP	*n*	CRP	*z*	*P*
<10 mg/L	76	3.12 (1.80,6.66)	-6.94	0.000^∗∗^
≥10 mg/L	59	23.14 (12.98,45.78)		

^∗∗^ *P* < 0.01.

**Table 4:** Area under the curve.

Test result variable(s)	Area	Asymptotic sig.^b^	Asymptotic 95% confidence interval		Cutoff value	Specificity	Sensitivity
			Lower bound	Upper bound			
WBC	0.685	0.000	0.594	0.775	8.9	0.688	0.655
Ca199	0.977	0.000	0.953	1.000	59.54	0.935	0.931

The test result variable(s): WBC has at least one tie between the positive actual state group and the negative actual state group. Statistics may be biased. ^a^Under the nonparametric assumption. ^b^Null hypothesis: true area = 0.5.

**Table 5:** Multiple linear regression for WBC.

Variables	Std. error	Standardized coefficients	*t*	*P*	95.0% confidence interval for B		Collinearity statistics	
		Beta			Lower bound	Upper bound	Tolerance	VIF
(Constant)	2.434		2.121	0.036	0.346	9.978		
Gender	0.897	0.043	0.502	0.617	-1.325	2.225	0.965	1.037
Age	0.033	0.129	1.495	0.137	-0.016	0.116	0.942	1.061
CA199	0.002	0.212	2.077	0.04	0	0.007	0.677	1.476
Tbil	0.007	0.034	0.33	0.742	-0.011	0.015	0.678	1.474

**Table 6:** Multiple linear regression for CRP.

Variables	Std. error	Standardized coefficients	*t*	*P*	95.0% confidence interval for B		Collinearity statistics	
		Beta			Lower bound	Upper bound	Tolerance	VIF
(Constant)	19.477		2.238	0.027	5.053	82.119		
Tbil	0.052	0.144	1.392	0.166	-0.031	0.175	0.678	1.474
Gender	7.18	-0.005	-0.054	0.957	-14.59	13.819	0.965	1.037
Age	0.268	-0.123	-1.4	0.164	-0.904	0.155	0.942	1.061
CA199	0.015	0.075	0.723	0.471	-0.018	0.04	0.677	1.476

## Data Availability

Raw data to support the results of this study can be obtained from the corresponding author upon request.

## Abbreviations

ERCP: endoscopic retrograde cholangiopancreatography
CA199: carbohydrate antigen 199
WBCs: white blood cells
CT: computed tomography.
